# Correction: Kovács, K.; Kovács, K.E. Using the Behavioural Regulation in an Exercise Questionnaire (BREQ–2) in Central and Eastern Europe: Evidence of Reliability, Sociocultural Background, and the Effect on Sports Activity. *Int. J. Environ. Res. Public Health* 2021, *18*, 11834

**DOI:** 10.3390/ijerph19031178

**Published:** 2022-01-21

**Authors:** Klára Kovács, Karolina Eszter Kovács

**Affiliations:** 1 Institute of Educational Sciences and Cultural Management, Faculty of Arts, University of Debrecen, 4032 Debrecen, Hungary; kovacs.klara@arts.unideb.hu; 2 Institute of Psychology, Faculty of Arts, University of Debrecen, 4032 Debrecen, Hungary

## Missing Funding

In the original publication [1], funding from the “**University of Debrecen Faculty of Humanities Scholarly Fund**” to “**Klára Kovács and Karolina Eszter Kovács**” was not included. Thus, the correct Funding statement is the following:

**Funding:** This research is project no. 123847, supported through National Research, Development and Innovation, financed from the K_17 application program, and published with the support of a János Bolyai Research Scholarship (2016–2019). The publication was supported by the University of Debrecen Faculty of Humanities Scholarly Fund.

The authors apologize for any inconvenience caused and state that the scientific conclusions are unaffected. The original publication has also been updated.
